# Editorial: Case reports in PET imaging 2023

**DOI:** 10.3389/fmed.2024.1455227

**Published:** 2024-07-10

**Authors:** Carmelo Caldarella, Matteo Bauckneht, Ramin Sadeghi

**Affiliations:** ^1^Nuclear Medicine Unit, Dipartimento di Diagnostica per Immagini e Radioterapia Oncologica, Fondazione Policlinico Universitario Agostino Gemelli IRCCS, Rome, Italy; ^2^Nuclear Medicine, IRCCS Ospedale Policlinico San Martino, Genoa, Italy; ^3^Department of Health Sciences (DISSAL), University of Genoa, Genoa, Italy; ^4^Nuclear Medicine Research Center, Mashhad University of Medical Sciences, Mashhad, Iran

**Keywords:** case report, PET-CT imaging, uncommon disease, diagnostic performances, ^18^F-FDG

In recent years, the increasing need for early diagnosis, precise disease staging/restaging and patient-tailored treatment has highlighted the benefits of using advanced hybrid Nuclear Medicine imaging, such as Positron Emission Tomography-Computed Tomography (PET-CT) in clinical practice. Its ability to simultaneously collect functional and anatomical information and to detect pathology without concurrent morphological alterations allows it to guide the clinical management of patients and is of increasing interest in clinical research. This Research Topic includes 14 case reports that highlight the role of PET-CT in patients with uncommon pathological conditions as a valuable tool for differential diagnosis, staging/restaging, guiding therapeutic strategy, or introducing possible developments in acquisition techniques that may be suitable for the critically ill patient.

The incidence of renal tumors has been increasing worldwide in recent years, with an estimated 400,000 new cases annually (1): the most common histotypes are renal clear cell carcinoma (RCC) and urothelial carcinoma. In this Research Topic, an uncommon case of Xp11.2 translocation/TFE3 gene fusion-associated RCC is presented in which ^18^F-FDG PET-CT was used to detect multiple retroperitoneal lymph nodes and was used to exclude disease recurrence and metastasis in long-term post-surgery follow-up (Huang, Peng et al.). ^18^F-FDG PET-CT may exhibit high uptake in other rare malignant kidney diseases, such as myxoid liposarcoma, a soft-tissue sarcoma associated with the chromosomal translocation t_(12:16)_ (q13:p11) (2, 3), which rarely arises predominantly in the kidney: in one case report, ^18^F-FDG PET-CT was used to stage primary renal myxoid liposarcoma, demonstrating disease extension beyond the kidney with pancreatic body involvement, and excluding distant metastases (Huang, Chao et al.).

Uncommon malignancies may pose a significant challenge due to unpredictable behavior and unusual metastatic spread, which in most cases is more extensive than seen on morphological imaging. It is well known that the extent and activity of metastatic involvement as shown by ^18^F-FDG PET-CT has an impact on treatment response, recurrence risk and, ultimately, patient prognosis (4–9). This is relevant for therapeutic schemes that include immune system-regulating drugs such as pembrolizumab or nivolumab. In this Research Topic, an exceptionally uncommon case of non-gestational choriocarcinoma is presented, in which ^18^F-FDG PET-CT confirmed metastatic spread to lungs, brain, bone, and retroperitoneal lymph nodes, with markedly increased uptake; the patient was treated with pembrolizumab, although with serious adverse effects and an inadequate response, leading to a rapid increase in the size of the lung and brain lesions, and ultimately to the patient's death only 5 months after diagnosis (Huang, Zheng, Bao et al.). In another paper, ^18^F-FDG PET-CT performed for restaging in an operated post-pubertal testicular teratoma revealed increased uptake at the edges of an irregular mass below the left renal hilum. The lesion exhibited low uptake in the center due to necrosis (a marker of aggressiveness), but no other disease sites were found. The patient was then re-operated, and despite initial PET-CT evidence of multi-site abdominal-pelvic progression after surgery, he showed a complete metabolic response on a subsequent PET-CT scan (Jiao et al.).

Attention should be paid to patients undergoing therapy with immune checkpoint inhibitors since pseudo-progression (increase in uptake/size by known lesions or appearance of new lesions and subsequent response) may complicate the evaluation of treatment efficacy. A case of unresectable liver recurrence of uveal melanoma treated with pembrolizumab, and long-term follow-up ^18^F-FDG PET-CT is published in this Research Topic. The patient ultimately showed a dramatic response to pembrolizumab after 28 months of therapy, despite three episodes of pseudo-progression which appeared on ^18^F-FDG PET-CT as increased uptake in the known liver lesion and the appearance of other liver lesions (Amrane et al.). This points to the need for new response criteria, such as iPERCIST and imPERCIST, which reduce the risk of overdiagnosis (10, 11).

PET-CT plays a role in the staging of rare malignancies by detecting distant diseases. In this Research Topic, a case of primary intracardiac diffuse large B-cell lymphoma is presented: the patient exhibited multiple nodular soft tissue density lesions in the heart and pericardium with increased ^18^F-FDG uptake, and multiple lymph nodes with a variable increase in ^18^F-FDG uptake in the mediastinum, left axilla and near the left kidney (Huang Z. et al.). Moreover, the distribution of an uncommon malignancy may mimic another more frequently observed condition, as illustrated by the case of a 57-year-old woman complaining of dizziness and headaches, with multiple sites of bone destruction in the skull, spine and pelvis on MRI, and multiple ^18^F-FDG-avid foci throughout the skeleton (including the sternum, bilateral clavicles, scapulae and ribs). This distribution, in the absence of a primary tumor, was consistent with multiple myeloma; however, histology of the most active skull lesion revealed a multicentric primary angiosarcoma of the bone, an exceptionally unusual occurrence (Huang, Xiao et al.). Moreover, an uncommon benign condition may be the first clinical manifestation of a common malignant disease, as in the case of a woman with retroperitoneal fibrosis and renal insufficiency, associated with a histologic finding of breast cancer metastasis in the right kidney: as morphological imaging failed to detect the primary lesion, ^18^F-FDG PET-CT was performed which revealed bilateral breast cancer (histologically confirmed as lobular carcinoma) with bone, lymph node and kidney metastases, thus underlying the advantages of functional imaging (Song et al.). The whole-body performance of PET-CT can also be highlighted in some benign conditions: in particular, in this Research Topic a case of sporadic schwannomatosis is reported, in which PET-CT revealed an uncommon involvement of the lumbar spinal canal, not seen on MRI, and a correlation between ^18^F-FDG uptake and histological characteristics (Li et al.).

Transplantation is a well-recognized treatment for many severe diseases and the only chance for survival when irreversible damage to vital functions occurs; however, immunosuppression is required to avoid rejection, which can lead to a wide spectrum of complications, either infectious, tumoral or non-neoplastic lymphoproliferative ones. In this context, two papers in this Research Topic address the role of ^18^F-FDG PET-CT in detecting and monitoring two rare post-transplant complications. Specifically, in a patient with blastic plasmacytoid dendritic cell neoplasm (BPDCN) involving the left cheek, lymph nodes and bone marrow, PET-CT performed after allogeneic hematopoietic stem cell transplantation and immunosuppressive therapy revealed multiple ^18^F-FDG-avid lymph nodes in the chest and abdomen, not seen on a pre-treatment scan, and the disappearance of known BPDCN lesions. A biopsy of the most ^18^F-FDG-avid mesenteric lesion revealed a post-transplant lymphoproliferative disorder. This demonstrated the potential of ^18^F-FDG PET-CT in detecting and monitoring such severe and exceptionally rare conditions, and in suggesting a lesion for biopsy (Chen et al.). The other case highlights the role of ^18^F-FDG PET-CT in detecting three complications that occurred sequentially in the same patient after renal transplantation: the first was a multifocal diffuse large B-cell lymphoma in the liver (without other disease sites on PET-CT); then, during chemotherapy for the lymphoma, an ^18^F-FDG-active bronchial thickening was revealed to be aspergillosis; finally, after successful antifungal treatment ^18^F-FDG PET-CT revealed a necrotizing granulomatous inflammation in the right vastus lateralis. Such complications would not have been detected without functional imaging (Huang, Zheng, Zhang et al.).

The liver is a typical site of metastasis, especially for gastrointestinal malignancies, whereas primary hepatic malignancies are less common and are mostly represented by hepatocellular carcinoma (HCC), accounting for more than 800,000 deaths annually (12). However, much more unusual pathological entities can occur in the liver, as reported in two articles in which PET-CT was useful for lesion characterization and staging. In particular, ^18^F-FDG PET-CT excluded distant disease in a patient with an incidentally found liver lesion suspected of being HCC; however, immunohistochemistry revealed that the lesion was a peri-vascular epithelioid tumor, a mesenchymal malignancy that only rarely involves the liver primarily (Yang et al.). Somatostatin receptor ligands are routinely used in PET-CT evaluation of patients with neuroendocrine tumors (NETs), with better performance than ^18^F-FDG due to more specific uptake and a higher lesion-to-background ratio; the liver is usually involved by metastases, while primary hepatic NETs are extremely rare (only 0.8% of all NETs) (13). In the case of a woman with an incidentally found liver mass, suspected of metastatic malignant tumor, ^18^F-FDG PET-CT showed uptake in two liver lesions and multiple lymph nodes in the hepatogastric ligament, therefore raising the suspicion of HCC. However, since A-fetoprotein was normal, [^18^F]Al-F-octreotide PET-CT, an emerging approach for somatostatin receptor functional imaging, was performed and showed even higher uptake than ^18^F-FDG in liver lesions and lymph nodes, which were subsequently confirmed as a primary hepatic NET with lymph node metastases (Zhang et al.).

Finally, recent advances in PET-CT technology, such as long-axial field-of-view (LAFOV) which result in a dramatic reduction in acquisition time and injected activity (14), may make PET-CT suitable for intensive care unit patients, as demonstrated in the case of Hemophilus influenza sepsis and pericarditis in which LAFOV allowed a 12-min whole-body scan revealing FDG uptake in the pericardial layers, pleura and right knee synovitis (van Snick et al.).

## Author contributions

CC: Conceptualization, Methodology, Supervision, Writing – original draft, Writing – review & editing. MB: Conceptualization, Methodology, Supervision, Writing – original draft, Writing – review & editing. RS: Conceptualization, Methodology, Supervision, Writing – original draft, Writing – review & editing.
